# Spinal muscular atrophy in Brazil: from individual treatment to global management^☆^

**DOI:** 10.1016/j.jped.2024.11.001

**Published:** 2024-11-13

**Authors:** Laurent Servais, Cristiane Araujo Martins Moreno

**Affiliations:** a Department of Paediatrics, University of Oxford, MDUK Oxford Neuromuscular Centre & NIHR Oxford Biomedical Research Centre, Oxford, UK; b Department of Paediatrics, University and University Hospital of Liège, Neuromuscular Reference Centre, Liège, Belgium; c Departamento de Neurologia, Faculdade de Medicina da Universidade de São Paulo (FMUSP), São Paulo, São Paulo, Brazil

Spinal muscular atrophy (SMA) is an autosomal recessive motor neuron disease. It presents clinically as progressive muscle weakness, and in the most severe and common cases, includes swallowing difficulties and respiratory insufficiency, often leading to death. Patients are classified into five groups, from 0 to 4, based on their maximum motor ability and age of onset, which can range from in-utero to adulthood. Recent data from the US and EU indicate that SMA affects 1 in 14,300 infants at birth^1^ A recent newborn screening program in the state of Minas Gerais, Brazil, identified 12 babies among 104,000 tested infants over six months, suggesting a potentially higher prevalence in Brazil. However, further follow-up would be necessary for stronger statements.^2^^,^^3^

In recent years, three disease-modifying treatments (DMTs) have been approved worldwide, including in Brazil. These treatments allow for dramatic improvement in lethality and motor function, especially when initiated early in the disease course. These drugs have all been studied in children receiving standard care, and the most recent guidelines emphasize the importance of multidisciplinary care alongside DMT to maximize their efficacy.^4^

In this issue of *Jornal de Pediatria*, Albuquerque et al.^5^ present a single-center cohort study of 81 patients living with SMA – both treated and untreated with DMT in Porto Alegre, Brazil. The overwhelming majority of treated patients received nusinersen, an intrathecally injected oligonucleotide that increases SMN protein production from the *SMN2* gene by modifying pre-mRNA splicing. Nusinersen was the first drug approved in Brazil and has since been followed by gene-replacement therapies, which six patients received, and the oral splicing modifier risdiplam, received by two patients. In the cohort, most SMA1 patients received treatment, compared to one-third of the SMA2 patients and 10 % of SMA3 patients. Consistent with findings in the literature, most patients experienced clinically significant improvements, although later treatment initiation and lower baseline motor function scores correlated with reduced therapeutic response. The clinical and genetic characteristics of the cohort were similar to those of other countries, except that SMA3 was the most common subtype, as previously observed in other Brazilian studies.^6^

It is remarkable to see a child affected by a lethal condition not only surviving but also achieving new motor milestones. Access to innovative treatments is a testament to a country's consideration for its most vulnerable citizens/children affected by a rare and severe disease. However, the impressive results observed in spinal muscular atrophy should prompt reflection on the sustainability and reproducibility of these developments. Albuquerque and colleagues highlighted that only half of the SMA2 patients in their study received respiratory therapy, pointing to limited access to non-pharmacological therapies in Brazil. This study illustrates the contrast between access to expensive innovative therapies and limited access to standard care within a specialized center in southern Brazil—a contrast that becomes even more pronounced when cost is considered. The official cost of nusinersen in Brazil is R$ 2.5 million for the first year, with an annual maintenance cost of R$ 1.3 million. Gene therapy costs R$ 8 million per single dose and judicialization^7^ of cases has given rise to a number of high-cost treatments delivered outside consensual clinical criteria. In comparison, a physiotherapy session costs between R$ 50.00 and R$ 500.00, depending on the region or insurance coverage, meaning that the healthcare cost of a single dose of gene therapy could provide physiotherapy three times per week for approximately 20 children over 20 years. The maintenance phase cost of nusinersen for a single child could cover physiotherapy for 86 infants. It is worth noting that these DMT costs exclude expenses related to drug administration, necessary ancillary exams, and management of adverse effects, all of which vary based on regional guidelines and safety profiles.

This new reality, as illustrated in the paper by Albuquerque et al., is not unique to Brazil but is common in many rapidly growing countries that represent attractive markets for the pharmaceutical industry but lack fully developed state-funded medical infrastructure.

Innovative treatment should not be seen as a substitute for standard care. All innovative treatments have been developed in children receiving standard care, and the absence of proper respiratory, nutritional, and physiotherapy management may negate the benefit of the expensive innovative therapies. Administration of DMT does not eliminate the need for multidisciplinary care.

Fortunately, there is a solution to avoid exponential cost increases. Several studies have shown a significant difference in treatment outcomes between children treated before and after symptoms onset.^8^ Simply put, patients with three copies of SMN2 identified at birth and treated immediately have a high likelihood of normal motor development. The oldest of these children, now around six to seven years old, are doing well.^1^ In contrast, similar patients identified by symptoms after a lengthy diagnostic journey generally present with classical SMA2, characterized by a lack of autonomous ambulation, scoliosis, and restrictive respiratory syndrome, which DMTs cannot reverse. Consequently, direct and indirect costs are significantly higher for patients treated after symptom onset.^9^ Findings reproduced across studies globally have spurred the development of several pilot and national newborn screening programs.^10^ Health economic evaluation confirms that SMA NBS is a highly cost-saving healthcare intervention in countries like Brazil, where DMT are available.^11^

Brazil, a country of around 200 million people (IBGE census), currently includes only six diseases in its National newborn screening program, though several pilot projects are evaluating SMA's inclusion. However, disease inclusion in the National program has historically been slow, in contrast to the urgent need for early intervention in affected patients.^2^^,^^3^

Given the incidence of 1 in 8400 and an annual birth rate of approximately 2.6 million, an estimated 309 children with SMA are born each year in Brazil. Without newborn screening, the additional costs for DMT and standard care—including physiotherapy, MDT management, ventilation, scoliosis management, and wheelchair^12^ could quickly become unsustainable. Additionally, the healthcare system's capacity to manage these patients with high needs and frequent respiratory infections will be increasingly strained.

In this context, NBS is an urgent medical, economic and ethical imperative. Developing standard care nationwide is also essential, as investing heavily in DMT is nonsensical if treated patients lack MDT and multi-professional management.

The journey of innovative therapies in SMA provides valuable lessons for similar situations that may arise with other conditions. Recent advances in treatments for Duchenne Muscular Dystrophy, Rett Syndrome, Angelman syndrome, and the approval of gene therapy for metachromatic leukodystrophy open new possibilities for patients with severe, progressive conditions.^13^^,^^14^ Through SMA, we have learned that treatment is not a cure and should not be presented as such to the community. Managing community expectations can help avoid crowdfunding and lottery-based fundraising, both ethically questionable mechanisms widely used in SMA. We have learned that successful clinical development relies on robust science, the availability of natural history data, and well-validated outcome measures. We have also recognized the importance of establishing standard care and implementing NBS before launching an innovative DMT. Finally, managing individual expectations is crucial to avoid requests to add or switch therapies in patients responding well, but below parental expectations.

By reporting real-world data from a tertiary Center in Brazil, Albuquerque et al. conveys the joy of seeing children survive a lethal disease like SMA1 and potentially become productive adults and, hopefully, happy individuals. However, their findings should also prompt us to confront our collective responsibility. The healthcare system must be effective for the greatest number of people, and in an era of high-cost therapies, the only way to achieve this is to proactively develop standard care and NBS programs, enabling DMT efficacy at an acceptable and sustainable cost-to-benefit ratio.

## Conflicts of interest

CAMM has nothing to declare. LS has given consultancy or lectures for Biogen, Roche, Novartis, BioHaven, Scholar Rock and Zentech.
